# Enhanced vascular inflammation in patients with advanced prostate cancer receiving hormone therapy

**DOI:** 10.1007/s00259-025-07409-6

**Published:** 2025-06-17

**Authors:** Holger Einspieler, Dina Muin, Ilva Kristiana Langrate, Stefan Schmitl, Clemens P. Spielvogel, Barbara J. Fueger, Xiang Li, Gero Kramer, Shahrokh F. Shariat, Marcus Hacker, Sazan Rasul

**Affiliations:** 1 Department of Biomedical Imaging and Image-Guided Therapy, Division of Nuclear Medicine, Medical University of Vienna, Währinger Gürtel 18-20, Vienna, 1090 Austria; 2 Department of Biomedical Imaging and Image-Guided Therapy, Division of General and Pediatric Radiology, Medical University of Vienna, Vienna, 1090 Austria; 3 Department of Urology, Comprehensive Cancer Center, Vienna General Hospital, Medical University of Vienna, Vienna, 1090 Austria; 4 Department of Urology, Weill Cornell Medical College, New York, NY 10065 USA; 5 Department of Urology, Second Faculty of Medicine, Charles University, Prague, 15006 Czech Republic; 6 Department of Urology, University of Texas Southwestern Medical Center, Dallas, TX 75390 USA; 7 Division of Urology, Department of Special Surgery, Jordan University Hospital, The University of Jordan, Amman, Jordan

**Keywords:** Prostate cancer, [^18^F]FDG, PET/CT, Hormone therapy, Arterial inflammation, Atherosclerosis

## Abstract

**Purpose:**

While blocking androgen production and action effectively slows prostate cancer (PCa) progression, it is associated with significant side effects, including an increased risk of cardiovascular disease. Inflammatory activity within atherosclerotic arteries can be assessed using [^18^F]FDG-PET imaging. Recently, [^18^F]FDG-PET has also gained relevance in PCa patients - alongside PSMA-targeted PET - for evaluating tumor aggressiveness. This study investigated the effect of hormone therapy on arterial inflammation in PCa patients using [^18^F]FDG-PET.

**Methods:**

Thirty-two PCa patients receiving hormone therapy were compared to 17 age-matched PCa patients who had not undergone hormonal treatment in the 12 months prior to imaging. All participants underwent [^18^F]FDG-PET/CT scans. Regions of interest (ROIs) were placed across several arterial segments, as well as in the superior vena cava (SVC), spleen, and bone marrow. To account for background vascular activity, blood-pool activity in the SVC was used for correction, and target-to-background ratios (TBRs) were calculated for each arterial segment. A semi-quantitative calcified plaque (CP) score was also recorded.

**Results:**

Patients receiving hormone therapy exhibited significantly higher TBR_max_ values in the abdominal aorta, ascending aorta, thoracic descending aorta, and in the combined analysis of all arteries (mean TBR_max_: 1.6 vs. 1.4; all *p* < 0.05). Similarly, TBR_mean_ values were significantly elevated in the abdominal and ascending aorta, as well as in the combined arterial analysis (all *p* < 0.05). No significant differences were observed between groups in age, BMI, total cholesterol, LDL, CRP, or CP scores (all *p* > 0.05).

**Conclusion:**

Advanced PCa patients undergoing hormone therapy demonstrate increased arterial inflammation on [^18^F]FDG-PET imaging compared to non-hormonally treated controls. These findings support a possible mechanistic link between hormone therapy and the elevated cardiovascular risk observed in this patient population.

## Introduction

Prostate cancer (PCa) is one of the most common malignancies and the second leading cause of cancer-related deaths in men worldwide [1]. Early detection and accurate staging are critical, as the 5-year survival rate varies significantly from nearly 100% in early-stage disease to approximately 30% in patients with advanced-stage cancer [2].

In patients with advanced PCa, hormone therapy, particularly androgen deprivation therapy (ADT), remains a cornerstone of treatment. ADT reduces or blocks the effects of androgen [3]. However, beyond its anti-tumor effects, ADT also impacts glucose homeostasis, lipid metabolism, and systemic inflammation [4], while diminishing the protective role of testosterone in maintaining endothelial function [5]. In later stages of the disease, androgen receptor pathway inhibitors (ARPIs) further enhance androgen blockade by directly targeting the androgen receptor signaling pathway. While both ADT and ARPIs are effective in slowing disease progression, they are associated with a range of adverse effects, notably an increased risk of cardiovascular (CV) disease [6]. Multiple studies have shown that hormone therapies are linked to a higher incidence of CV events, including hypertension, acute coronary syndromes, cardiac arrhythmias, and venous thromboembolism [7, 8].

A major contributor to CV disease is atherosclerosis, a condition characterized by the buildup of lipid-rich plaques along the arterial endothelium. Central to the development and progression of atherosclerosis is chronic vascular inflammation [9]. This inflammatory activity can be visualized non-invasively using [^18^F]fluorodeoxyglucose positron emission tomography ([^18^F]FDG-PET), which enables detection of metabolically active inflammatory cells within the arterial wall. In this context, the Cardiovascular Committee of the European Association of Nuclear Medicine has proposed standardized protocols for atherosclerosis imaging using [18F]FDG-PET, emphasizing its value in both clinical and research settings [10].

Parallel to its application in vascular imaging, [^18^F]FDG-PET has also gained importance in the management of PCa. In patients with metastatic castration-resistant prostate cancer (mCRPC), dual-tracer imaging using both [^68^Ga]PSMA and [^18^F]FDG-PET has been shown to aid in assessing disease heterogeneity and predicting response to PSMA-directed radioligand therapy (RLT) [11, 12]. The combined use of these imaging modalities improves lesion detection and can identify aggressive tumor phenotypes that may be less responsive to PSMA-targeted approaches.

Given the established association between hormone therapy and increased CV risk, and the utility of [^18^F]FDG-PET in assessing arterial inflammation, the aim of this study was to investigate whether hormone therapies in PCa patients are associated with increased FDG uptake in arterial vessels. Increased FDG uptake may serve as a surrogate marker of arterial inflammation and provide further insight into the mechanisms linking hormonal treatment and CV disease risk.

## Methods

### Patients

A total of 65 patients with metastatic PCa, who underwent [^18^F]FDG PET/computed tomography (CT) at the Vienna General Hospital between June 2022 and March 2024, were initially considered for this retrospective study. Sixteen patients with a history of chemotherapy or radioligand therapy prior to the PET scan were excluded, yielding a final cohort of 32 patients receiving hormone therapy at the time of the scan (cohort A) and 17 age-matched patients who had not received hormone therapy for at least 12 months prior to the scan (cohort B). Laboratory parameters relevant to this study, including C-reactive protein (CRP), total cholesterol, low-density lipoprotein (LDL), and fasting blood glucose were measured using hospital conventional assays. Additional demographic factors were also collected.

This study was approved by the Ethics Committee of the Medical University of Vienna (EK: 1745/2021). Due to its retrospective design, written informed consent was not required from the included patients for data collection and analysis.

### PET/CT examination

All [^18^F]FDG-PET/CT scans were performed using a multi-detector integrated PET/CT device (Siemens Biograph128 Vision Quadra Edge, Erlangen, Germany) with a 106 cm FOV. Whole-body scans were conducted from the skull to the thigh 45–60 min after the injection of approximately 3 MBq/kg body weight of [^18^F]FDG. CT scans were obtained with the following parameters: 80–140 kV and 80–180 mAs; slice thickness 2 mm; matrix 512 × 512. CT imaging was performed with the patient in a shallow breathing position to ensure alignment with the PET slices. Contrast medium was administered unless contraindicated. The PET acquisition protocol was conducted directly after CT imaging. PET examinations were performed with a duration of 4 min in one position, using iterative reconstruction with a point-spread-function-based algorithm. Subsequently, scatter and attenuation corrections were then applied based on the CT scan, with a PET matrix of 220 × 220. The PET voxel size was 1.65 × 1.65 × 2 mm.

### Image analysis

**Fig. 1:**
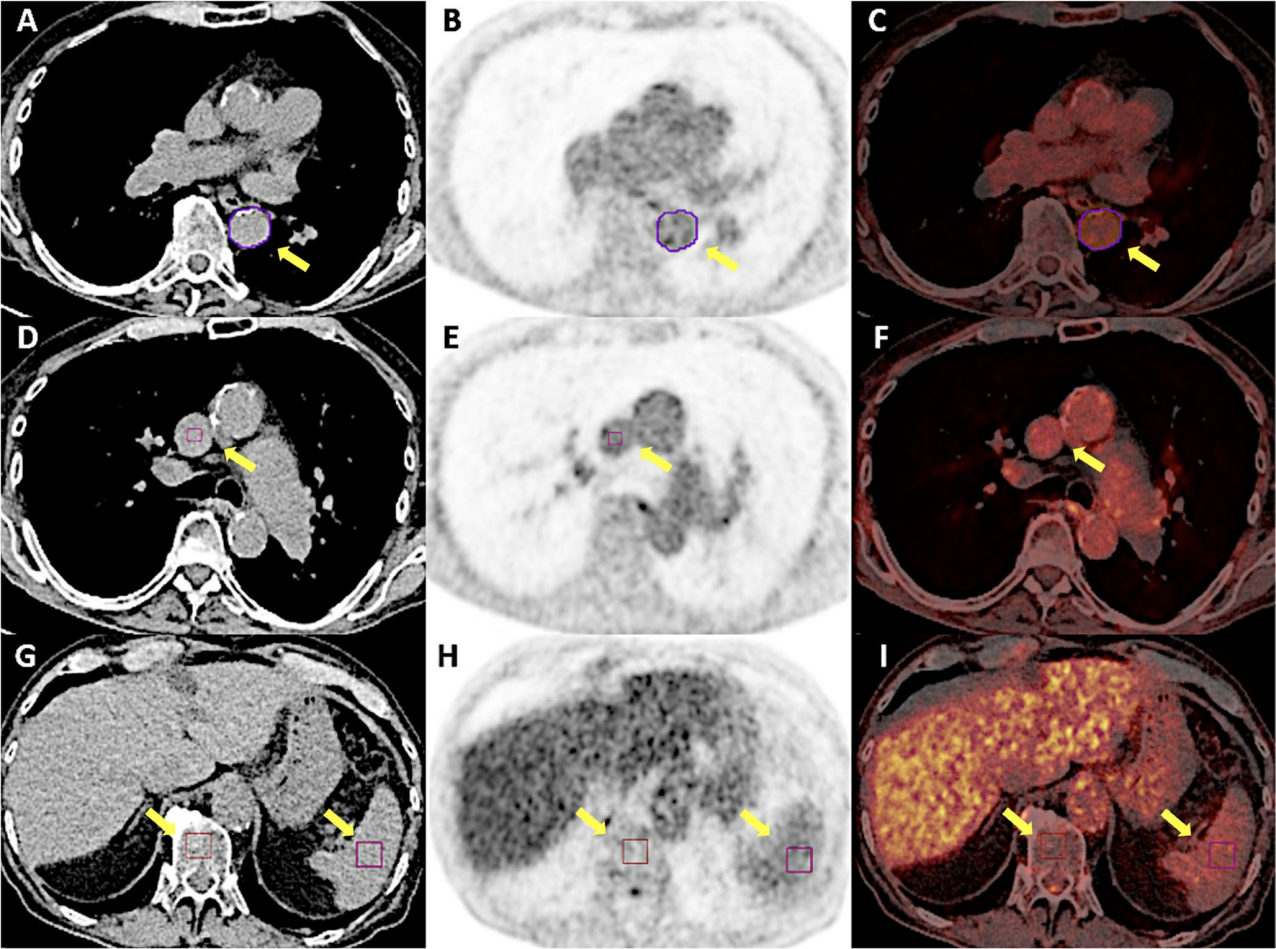
[^18^F]FDG-PET/CT image analysis: An example of image analysis is shown using transaxial CT (A, D, G), PET (B, E, H), and fused [¹⁸F]FDG-PET/CT (C, F, I) images. A circular ROI was manually drawn around the thoracic descending aorta (**A**–**C**) on the CT images, and SUV_mean_ and SUV_max_ values were extracted from the corresponding PET images using this ROI. Additionally, cuboidal VOIs were placed in the superior vena cava (**D**–**F**), spleen, and bone marrow (**G**–**I**) for each patient

A dedicated workstation with Hybrid 3D software (version 4.17, Hermes Medical Solutions, Stockholm, Sweden) was used for image analysis. PET image intensities (Bq/mL) were converted to standardized uptake values (SUV). Two experienced nuclear medicine physicians determined the mean (SUV_mean_) and the maximum (SUV_max_) values in several vessels and organs. They manually draw a region of interest (ROI) in the abdominal aorta, ascending aorta, aortic arch, thoracic descending aorta and carotid artery, encompassing the entire artery, including the arterial wall and lumen. They also placed a cuboidal volume of interest (VOI) with a target size of 1.60 mL in the superior vena cava (SVC) and with a target size of 13.50 mL in the spleen and bone marrow (Fig. 1). In all patients, ROIs were excluded if there was suspected spill-in of activity from nearby structures or suspicious metastatic lesions. Finally, PET parameters were normalized to the blood pool by dividing the SUV_mean_ and SUV_max_ of each arterial site and organ by the SUV_mean_ of the VOI placed in the SVC, to obtain target-to-background ratios (TBRs).

The CT component of the [^18^F]FDG PET scan was used to analyze the presence of calcified plaque (CP) in the different arterial segments. The extent of calcification was assessed semi-quantitatively using a scale based on a previously published study [13]: 0 = absence of calcified plaque in the vessel circumference; 1 = small plaques covering < 10% of the vessel circumference; 2 = plaques involving 10–25% of the vessel circumference; 3 = plaques covering 25–50%; 4 = plaques exceeding 50% of the vessel circumference.

### Statistical analysis

Statistical analysis was performed with IBM Mac SPSS Version 28. Descriptive variables were expressed as mean ± standard deviation (SD), or as median (minimum; maximum), or as absolute numbers (n). Group comparability of the two cohorts was assessed by comparing the baseline demographics. Normality and heteroscedasticity of continuous data were assessed and calculated using the Shapiro–Wilk test and the Levene test. The χ^2^ test was used when comparing categorical variables across cohorts, while the Fisher-Freeman-Halton exact test was applied in cases with small expected counts. Due to the small group size, the Mann–Whitney U test was used to compare continuous outcomes. 95% confidence intervals (CI) were calculated using the Hodges-Lehmann estimator. A *p*-value of < 0.05 was determined to be statistically significant. Correlations were calculated using Spearman’s rank correlation coefficient. Missing data were addressed using pairwise deletion in this study, excluding only cases with missing values for the relevant variables.

In addition, box plots were generated to illustrate the different SUV values of all vessels in both cohorts.

## Results

### Patient population

Demographic characteristics of both cohorts are demonstrated in Table 1. No significant differences were found in age, BMI, or any relevant blood parameter among the two study groups. In hormone therapy patients, the most common medication was Leuprorelinacetat (*n* = 18), followed by Abiraterone (*n* = 8), Degarelix (*n* = 8), Enzalutamide (*n* = 4), Apalutamide (*n* = 3), Triptorelin (*n* = 1) and Darolutamide (*n* = 1).

Patients in cohort A had received continuous hormone therapy for PCa for an average of 5.4 years at the time of the [^18^F]FDG-PET examination. In contrast, patients in cohort B (*n* = 17) had not received any hormone therapy in the last 12 months prior to PET imaging.

**Table 1:** Demographic characteristics of both cohorts with PCa

Parameters	Cohort A (*n*:32)	Cohort B (*n*:17)	*p*-value
Age in years (mean ***± ****SD*)	70.9 (*± 8.0)*	71.3 (*± 7.7*)	*p* = 0.83
BMI (mean ***± ****SD)*	26.5 (*± 5.4)*	26.0 (*± 3.8)*	*p* = 0.96
ECOG performance status:			
0 1 2	12 (37.5%) 16 (50.0%) 4 (12.5%)	9 (52.9%) 8 (47.1%) 0 (0.0%)	*p* = 0.327
Blood parameters (mean ***± ****SD*):			
Total cholesterol (mg/dL)	168.9 (± 53.0)	160.9 (± 38.9)	*p* = 0.67
LDL (mg/dL)	87.3 (± 34.0)	87.4 (± 31.7)	*p* = 0.50
CRP (mg/dL)	1.9 (± 2.2)	1.3 (± 2.2)	*p* = 0.29
Glucose (mg/dL)	121.2 (± 20.7)	125.9 (± 40.2)	*p* = 0.82
Current hormone therapies (n):	32	0	
Abiraterone (Zytiga^®^) Enzalutamide (Xtandi^®^)	8 4	0 0	
Degarelix (Firmagon^®^) Leuprorelinacetat (Eligard^®^) or Trenantone^®^) Apalutamide (Erleada^®^) Triptorelin (Pamorelin^®^) Darolutamide (Nubeqa^®^)	8 18 3 1 1	0 0 0 0 0	NA
Arterial hypertension (n):	20	9	*p* = 0.52
Other cardiovascular diseases (n)	7	4	*p* = 0.90
Smoker (n)	3	4	*p* = 0.18
Diabetes mellitus (n)	3	5	*p* = 0.07
Statins (n)	10	6	*p* = 0.77

*n* Number of patients; *SD* Standard deviation; *BMI* Body mass index; *ECOG* Eastern Cooperative Oncology Group; *NA* Not applicable

### Comparison of TBR_mean_ - and TBR_max_ -values in both cohorts

**Fig. 2:**
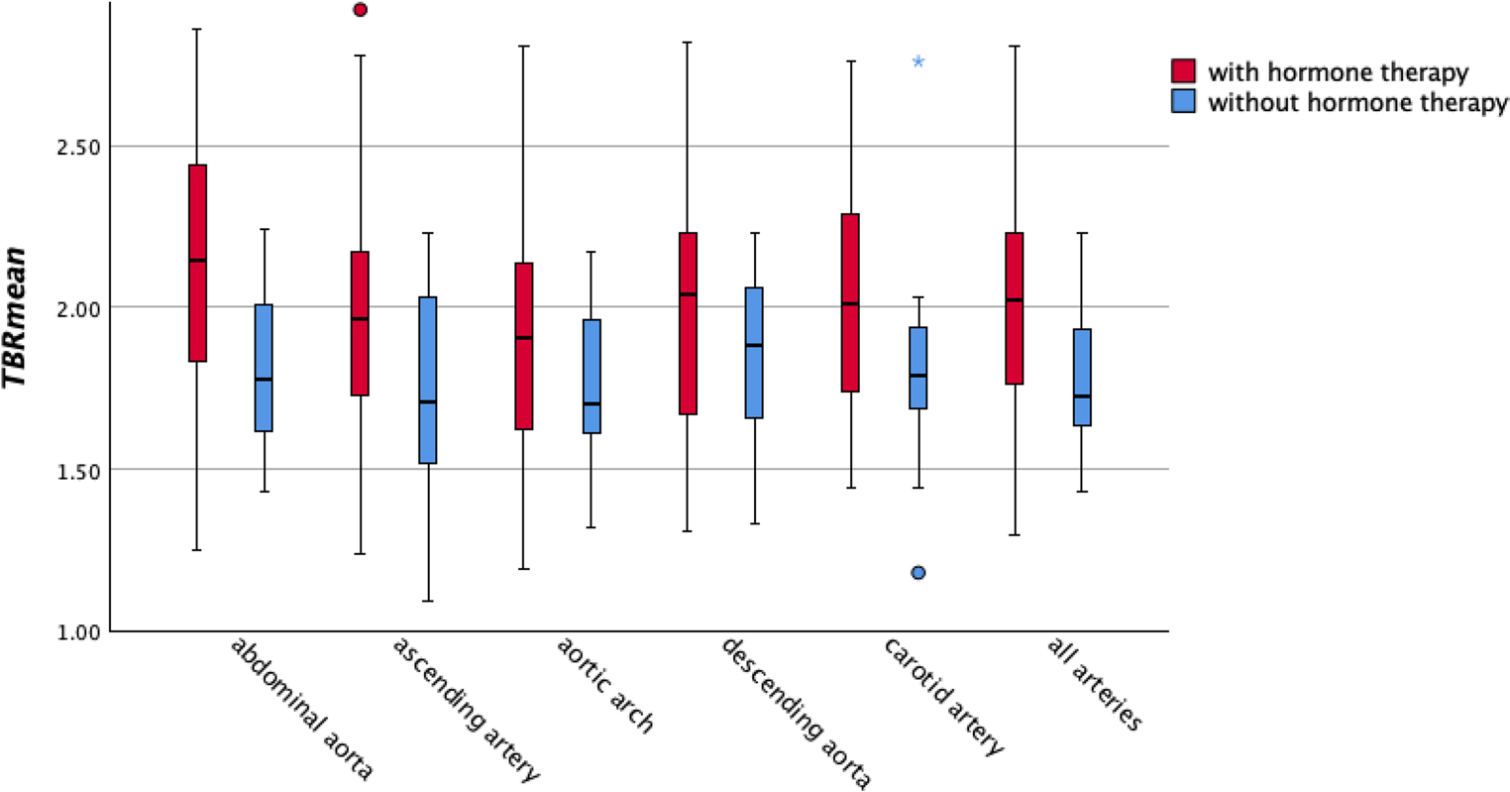
Boxplots with TBR_mean_ values of all included arteries of both studied cohorts: Boxplots showing TBR_mean_ values for the abdominal aorta, ascending aorta, descending aorta, aortic arch, carotid artery, and all arterial sites combined. Circles (○) represent outliers (> 1.5×IQR from the quartiles), and stars (★) indicate extreme outliers (> 3×IQR)

As displayed in Table 2; Fig. 2, patients on hormone therapy showed higher [^18^F]FDG-uptake of TBR_mean_ in the abdominal aorta (95% CI: 0.019–0.208, *p* = 0.019) and ascending aorta (95% CI: 0.008–0.158, *p* = 0.021).

**Table 2:** [^18^F]FDG PET parameters of both investigated cohorts with PCa

	Cohort A (*n*:32)		Cohort B (*n*:17)	
Localization	TBR_mean_^$^:	TBR_max_^$^:	TBR_mean_^$^:	TBR_max_^$^:
Abdominal aorta	1.1* (1.0; 2.0)	1.6* (1.2; 2.7)	1.0* (0.8; 1.4)	1.4* (1.1; 2.3)
Ascending aorta	1.1* (0.9; 1.7)	1.6* (1.0; 2.0)	1.0* (0.6; 1.2)	1.4* (1.2; 2.0)
Aortic arch	1.0 (1.0; 1.4)	1.6 (1.3; 2.6)	1.0 (0.8; 1.3)	1.5 (1.0; 2.0)
Thoracic descending aorta	1.1 (1.0; 1.4)	1.6* (1.3; 2.9)	1.0 (0.9; 1.3)	1.4* (1.2; 2.0)
Carotid artery	1.1 (0.8; 1.8)	1.4 (1.0; 2.2)	1.0 (0.6; 1.6)	1.2 (0.7; 1.9)
All arteries	1.1* (1.0; 1.6)	1.6* (1.3; 2.4)	1.0* (0.8; 1.3)	1.4* (1.2; 1.9)
Spleen	1.1 (1.0; 2.2)	1.6 (1.2; 2.9)	1.1 (0.9; 1.5)	1.5 (1.1; 2.1)
Bone marrow	0.8 (0.5; 1.6)	1.7 (1.0; 3.1)	0.9 (0.5; 1.3)	1.7 (0.9; 2.7)

*n* Number of patients; *TBR* Target to background ratio; * Significant differences (*p* < 0.05) between patients on hormone therapy and patients without hormone therapy at the time point of the [^18^F]FDG PET-scan; $ Values presented as median (minimum; maximum)

**Fig. 3:**
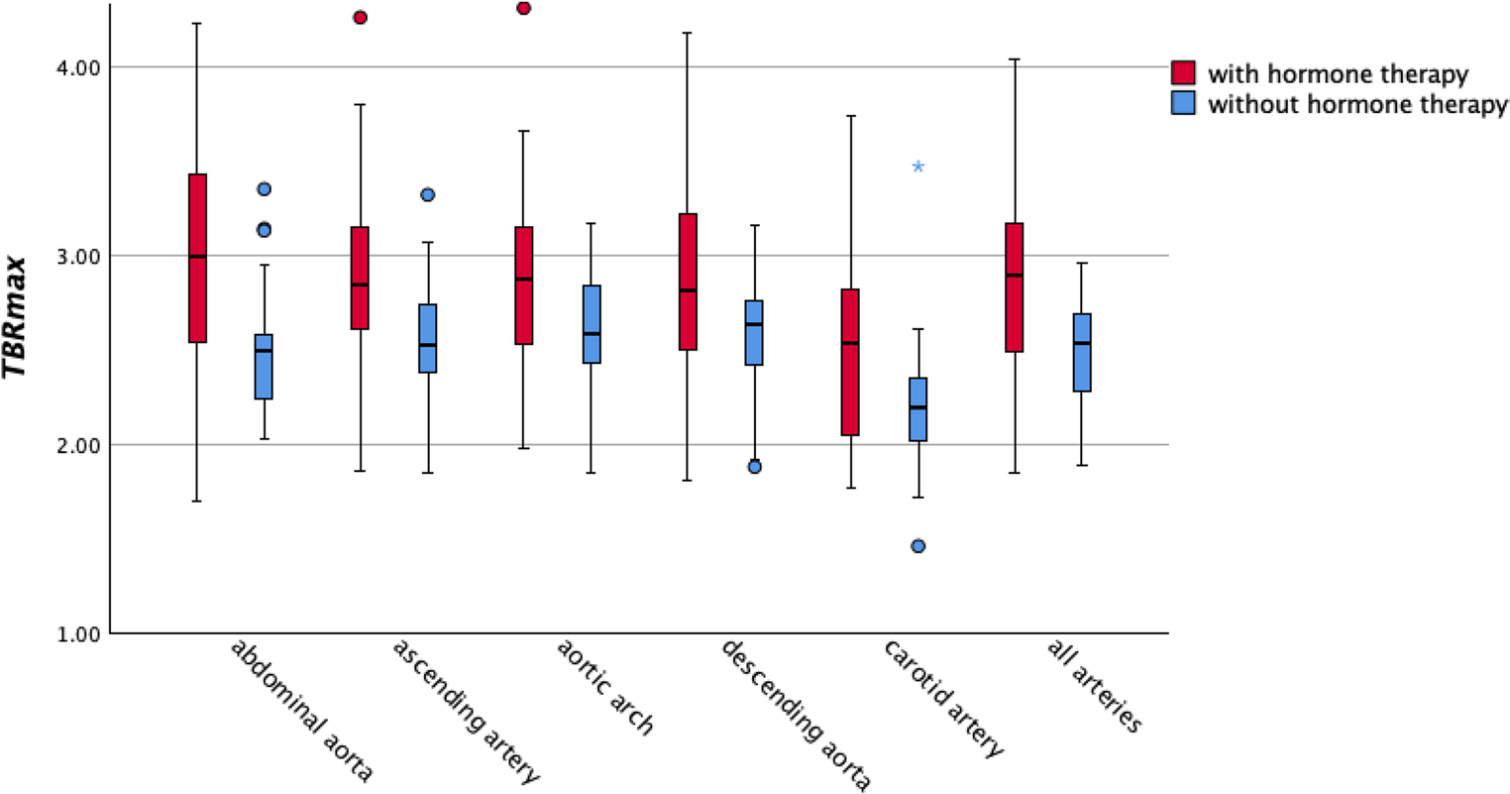
Boxplots with TBR_max_ values of all included arteries of both studied cohorts: Boxplots showing TBR_max_ values for the abdominal aorta, ascending aorta, descending aorta, aortic arch, carotid artery, and all arterial sites combined. Circles (○) represent outliers (> 1.5×IQR from the quartiles), and stars (★) indicate extreme outliers (> 3×IQR)

Furthermore, cohort A demonstrated significantly higher values of TBR_max_ in the abdominal aorta (95% CI: 0.063–0.350, *p* = 0.007), ascending aorta (95% CI: 0.014–0.227, *p* = 0.026) and thoracic descending aorta (95% CI: 0.014–0.250, *p* = 0.035), as demonstrated in Table 2; Fig. 3.

Finally, when all arteries were combined, significantly higher TBR-values were found in patients under hormone therapy as compared to those without (TBR_mean_ 95% CI: 0.008–0.163, *p* = 0.031; TBR_max_ 95% CI: 0.048–0.241, *p* = 0.006). No statistical different TBR_mean_- and TBR_max_-values were found between both groups in the spleen and in the bone marrow (all *p* > 0.05), as can be seen in Table 2.

### Assessment of calcification

The CP score was used for the assessment of calcification status in order to identify any discrepancies in the extent of CPs between the two cohorts.

The highest mean CP scores were observed in the abdominal aorta of patients with and without hormone therapy (3.0, 3.4; respectively), the lowest mean CP scores in the ascending aorta (0.7, 0.4; respectively). No statistically significant differences could be found when the mean sum of the CP scores of the two cohorts was compared (*p* = 0.951). Additionally, no statistically significant differences in CP scores were found in the abdominal-, ascending-, descending aorta, aortic arch or carotid arteries between the two cohorts (*p* > 0.05).

### Correlation of FDG uptake and calcification

To assess whether changes in FDG uptake were associated with the extent of calcified plaques, correlation analyses were performed between the CP scores and TBR-values in each arterial segment as well as in all segments combined. These analyses were conducted separately for cohorts A and B, as well as for the overall study population. No statistically significant correlations were found between TBR-values and CP scores across all arterial segments and patient cohorts (all *p* > 0.05). The observed correlation coefficients were consistently low (all *r* < 0.3).

## Discussion

In this study, we compared arterial inflammatory activity, as measured by [^18^F]FDG uptake, between age-matched PCa patients with and without ongoing hormone therapy. We observed significantly higher [^18^F]FDG uptake in the abdominal aorta, ascending aorta, and descending thoracic aorta among patients receiving hormone therapy, indicating increased arterial inflammation in this group.

Atherosclerosis is well established as a chronic inflammatory disease of the arterial wall [9]. Increased [^18^F]FDG uptake in vascular walls has been widely validated as a surrogate for inflammatory activity, correlating with plaque burden and risk of CV events [13, 14]. Prior studies have demonstrated that symptomatic atherosclerosis and vascular inflammation, particularly in the carotid arteries, are associated with elevated [^18^F]FDG uptake [15]. Given its prognostic relevance, [^18^F]FDG PET imaging is increasingly used to quantify arterial inflammation in patients across various disease states. For example, Boswijk et al. reported a correlation between [^18^F]FDG uptake and vascular inflammation in patients with thyroid carcinoma under TSH suppression [16], while Geraldino-Pardilla et al. observed similar findings in patients with rheumatoid arthritis [17] and Emami et al. in patients after acute coronary syndrome [18]. These studies underscore the utility of [^18^F]FDG-PET as a non-invasive tool to assess arterial inflammation and potential CV risk [13]. Our findings add to this body of evidence, demonstrating that hormone therapies, whether ADT or ARPI, are associated with increased [^18^F]FDG activity in the arterial wall in PCa patients. This supports the hypothesis that these treatments may contribute to heightened vascular inflammation and, consequently, a higher risk of CV events.

This observation aligns with prior epidemiologic evidence linking hormone therapy in PCa to increased CV morbidity and mortality. Jonušas et al. analyzed data from over 13,000 PCa patients and reported a significantly elevated risk of CV-related death, especially from ischemic heart disease and stroke, in those treated with ADT compared to non-users [19]. Similarly, a recent systematic review and meta-analysis of 24 randomized clinical trials found that ARPI therapy significantly increased the risk of CV events and CV-related mortality [20]. These findings emphasize the need for CV risk monitoring in patients receiving hormonal treatment for PCa.

Although CRP levels did not differ significantly between the two groups in our study, a tendency toward higher CRP concentrations was observed among hormone-treated patients (1.9 mg/dL vs. 1.3 mg/dL; *p* = 0.29). Previous studies have demonstrated a strong association between elevated CRP, increased arterial [^18^F]FDG uptake, and future CV events [21, 22].

Although hematopoietic organs have been partially observed to correlate with arterial inflammation and [^18^F]FDG uptake in literature [18, 23], we did not find statistically significant differences in our study. This suggests that the increased FDG uptake induced by hormone therapies may primarily reflect a localized inflammatory response in the vasculature, with possibly only a subtle systemic involvement that might become evident in larger cohorts, as hinted by the slight trend towards higher CRP concentrations in patients under hormone therapies, rather than a robust systemic immune stimulation.

In our study, we did not observe statistically significant correlations between CP scores and TBR-values, nor did we find relevant differences in CP scores at any arterial site when comparing the two cohorts. These results suggest that arterial inflammation, as determined by [^18^F]FDG uptake, may not be directly linked to the extent of calcified plaques in this patient population. Calcified plaques represent advanced, long-standing stages of atherosclerosis, whereas [^18^F]FDG uptake reflects more dynamic inflammatory activity [24]. Therefore, the lack of association in our study could indicate that the increased vascular inflammation observed may be related to earlier stages of plaque development, such as foam cell formation or to inflammatory processes, that are independent of calcification [25]. Moreover, differences in calcified plaque burden typically develop over longer periods and may not be significantly affected by the duration of hormonal therapy in our study.

Interestingly, total and LDL cholesterol levels were generally lower in our patient cohorts compared to the age-matched Austrian population, despite less than half of the patients receiving statin therapy [26]. This observation may reflect a catabolic state in some patients, as has been described in advanced cancer or systemic illness [27].

However, several limitations of this study must be acknowledged. The small sample size and retrospective design limit statistical power and generalizability. Furthermore, the absence of longitudinal data on actual CV outcomes restricts our ability to establish a causal link between increased arterial [^18^F]FDG uptake and clinical CV events. Lastly, while the two groups were age-matched, the lack of intra-individual (pre-/post-therapy) comparisons limits the precision of observed differences. Future prospective studies with larger cohorts are needed to further evaluate the vascular effects of specific hormone therapies using [^18^F]FDG PET. Identifying patients at highest CV risk could help tailor treatment strategies and support preventive interventions. As highlighted by Corona et al., the increased CV risk associated with ADT underscores the importance of pre-treatment screening and continued monitoring during therapy [28].

## Conclusion

This retrospective study suggests that prostate cancer patients undergoing hormone therapy exhibit higher arterial [^18^F]FDG uptake, indicating increased vascular inflammation and a potentially elevated risk of developing atherosclerosis and CV disease. These findings reinforce the need for CV risk assessment in patients treated with ADT or ARPI. Larger, prospective studies are warranted to validate these findings and clarify the impact of specific hormone therapies. Incorporating CV screening before and during hormone therapy may be critical to prevent major adverse CV events in this patient population.

## Data Availability

The datasets generated and/or analyzed in this study are available from the corresponding author upon reasonable request.
